# Musical hallucinations, secondary delusions, and lack of insight: results from a cohort study

**DOI:** 10.3389/fpsyt.2023.1253625

**Published:** 2023-09-28

**Authors:** Milou A. Buijk, René F. Lauw, Jan Adriaan F. Coebergh, Ouarda Bouachmir, Mascha M. J. Linszen, Jan Dirk Blom

**Affiliations:** ^1^Parnassia Psychiatric Institute, The Hague, Netherlands; ^2^Department of Neurology, Ashford/St. Peter’s Hospital, Chertsey, United Kingdom; ^3^Department of Neurology, St. George’s Hospital, London, United Kingdom; ^4^Department of Psychiatry, University Medical Center Groningen, Groningen, Netherlands; ^5^Faculty of Social and Behavioural Sciences, Leiden University, Leiden, Netherlands

**Keywords:** antipsychotic, cognitive impairment, dementia, hearing impairment, deafferentation, paranoia, risperidone, sensory deprivation

## Abstract

**Introduction:**

Although musical hallucinations do not tend to be accompanied by delusions, occasionally patients persistently accuse others of being responsible for causing the music they perceive, sometimes with severe social consequences such as frequently calling the police or moving house. In this study we seek to broaden our understanding of this rare type of musical hallucination that comes with secondary delusions and lack of insight, and to explore associations, underlying mechanisms, and treatment possibilities.

**Methods:**

The present study is part of a cohort study on musical hallucinations carried out in the Netherlands from 2010 through 2023. Participants underwent testing with the aid of the *MuHa Questionnaire*, *Launay-Slade Hallucinations Scale* (LSHS), *Schizotypal Personality Questionnaire* (SPQ), *Hamilton Depression Rating Scale* (HDRS), and *Mini Mental State Examination* (MMSE). Additionally, they underwent a brain MRI, electroencephalogram, and audiological testing.

**Results:**

Five patients out of a group of *N* = 81 (6%) lacked insight and presented with secondary delusions regarding the perceived music. They were all female, of advanced age, and hearing-impaired, and were diagnosed with cognitive impairment. In three patients (60%), risperidone was started. This had a positive effect on the hallucinations *and* secondary delusions.

**Conclusion:**

The pathophysiological process underlying musical hallucinations is multifactorial in nature. We consider cognitive impairment the most likely contributing factor of the secondary delusions and lack of insight encountered in our patients, and antipsychotics the most beneficial treatment. On the basis of these small numbers, no definite conclusions can be drawn, so further research is needed to elucidate the underlying mechanisms and to develop evidence-based treatment methods for people experiencing this rare and debilitating combination of symptoms. Since the black box warning of risperidone cautions against the use of this drug in elderly persons with dementia, a proper comparison with the efficacy and safety of other antipsychotics for this group is paramount.

## 1. Introduction

Musical hallucinations are characterized by melodies, tunes, songs, timbres, harmonics and/or rhythms, without the presence of an external source (1). Reliable incidence and prevalence figures are unknown, but in groups of people with a psychiatric illness and in those with an age of ≥65 years it is estimated that their incidence lies around 1 per 10,000 (2). Among elderly people with audiological complaints, their lifetime prevalence rate has been reported to range from 0.86 to 2.5% (3, 4). In general hospital settings, the point prevalence has been estimated to be 0.16% (5), and among adults with hearing impairment an incidence was found of 5.2% (6). Surveys in clinical populations suggest that musical hallucinations may occur more frequently in people diagnosed with schizophrenia (7) or obsessive-compulsive disorder (8). Other risk factors include social isolation, tinnitus, and hearing impairment, although these may not be independent factors (4). According to Saba and Keshavan (7), and an earlier study by Berrios (1), most people rate their musical hallucinations as positive. This may be true in comparison to the derogatory voices often reported in schizophrenia spectrum disorders, but later studies have shown that musical hallucinations also tend to be rated as intrusive and distressing (9), especially when they lead to or are comorbid to anxiety, depression, and impaired functioning. Even if this is not the case, people are often afraid of being (perceived as) psychiatrically ill, and may have difficulty concentrating and falling asleep (3, 5, 10). The pathophysiology of musical hallucinations is still in need of further elucidation. Some studies indicate the involvement of the superior temporal sulcus [e.g., (11)], while positron emission tomography (PET) studies indicate the involvement of the posterior part of the temporal lobes, right basal ganglia, inferior frontal cortices, cerebellum, pons, brainstem, and thalamus (12). Another study found activity in the left anterior superior temporal gyrus, motor cortex, posteromedial cortex, and left lateral orbitofrontal cortex (13), but the network involved in their mediation may well be more widely disseminated, and even include visual and motor areas (14).

In most cases, people who experience musical hallucinations have insight into the nature of their disorder. It is not uncommon for them to be surprised when they first find out that the music has no external source, but they usually accept within several days that it must be hallucinatory in nature (12), either by themselves or are after discussion with someone else. However, occasionally they may go on to develop delusions with a paranoid character, persistently accusing others of being responsible for it (15). This may have severe social consequences, such as frequently calling the police or moving house. For example, Bieler et al. (16) described a 92-year-old cognitively intact woman with hearing impairment and musical hallucinations who called the police because “her neighbors refused to turn off the music.” The hallucinations disappeared after a plug of cerumen and wool had been removed from her right ear, and they were attributed to the auditory Charles Bonnet syndrome, a variant of musical hallucinosis due to hearing loss (17). Another published case involves a 78-year-old woman with hearing loss, cognitive dysfunction, erotomanic delusions, and complex musical hallucinations, who heard her neighbor singing a familiar church song that was interrupted by provocative and sexually laden comments (18). The authors diagnosed her with dementia with psychotic symptoms, which reduced slightly with a combined treatment with clozapine, zonisamide, donepezil, and memantine. A third example stems from Mori et al. (19), involving a 73-year-old woman with Alzheimer’s disease who experienced familiar tunes that she kept attributing to her neighbors.

The aim of the present study is to broaden our understanding of this rare type of musical hallucination that presents with delusions and lack of insight, and to explore associations and potentially underlying mechanisms. To that end we draw on a prospective cohort study (to our knowledge the largest thus far) on musical hallucinations.

## 2. Materials and methods

The present study is part of a cohort study on musical hallucinations that was carried out from 2010 through 2023 at Parnassia Psychiatric Hospital, The Hague, in collaboration with the Haga Hospital. In 2010 the study received ethical approval from the Ethical Review Board of the Haga Hospital, The Hague (number: 10–114). Potential participants were referred to a specialized psychiatric outpatient department from all over the Netherlands by their mental health care provider, neurologist, otorhinolaryngologist, or general practitioner. All participants provided signed informed consent. They underwent testing with the aid of the *MuHa Questionnaire* (a tailor-made, yet non-validated questionnaire for the systematic assessment of musical hallucinations, see Supplementary material), and Dutch translations of the *Launay-Slade Hallucinations Scale* [LSHS; (20)], the *Schizotypal Personality Questionnaire* [SPQ; (21)], the *Hamilton Depression Rating Scale* [HDRS; (22)], and the *Mini Mental State Examination* [MMSE; (23)]. For the present study, the results of the questionnaires that are relevant will be discussed. In addition, we administered brain imaging (mostly brain MRI), electroencephalography (EEG), and audiological testing. When possible, we collected additional clinical information about the course and treatment of the hallucinations through the referring physicians. Upon inclusion, patients were followed up once a year to assess the course of their musical hallucinations with the aid of the *MuHa Questionnaire*. For the purpose of the present study, lack of insight was defined as the absence or limited recognition by the patient that their musical hallucinations are not originating from external sources, but are rather generated within their own mind. This lack of insight can lead the patient to mistakenly believe that the music they are hearing is real and originating from external sources, even though it is a product of their own mental processes. In accordance with this definition, lack of insight was assessed by reviewing the results of the *MuHa Questionnaire*, the intake reports (which included a comprehensive medical history), the psychiatric examination, and the collateral history.

## 3. Results

The total number of patients included in our study was 81. All of them were screened for secondary delusions and lack of insight by reviewing the results of the *MuHa Questionnaire* and intake reports which included a comprehensive medical history, psychiatric examination and collateral history. Five elderly patients (6%) fitted this profile.

### 3.1. Patient 1

In 2011, a 76-year-old woman with a history of dependent personality disorder, impaired hearing, and tinnitus was transferred from her nursing home to Parnassia Psychiatric Institute due to auditory hallucinations with delusional interpretations, resulting in behavioral problems. She heard bilateral buzzing, tones, and songs. The hallucinations were present daily, from awakening until late at night and she experienced them as bothersome. The hallucinations hindered her ability to fall asleep (for which she used oxazepam), and disrupted her concentration and conversations. The patient thought that the music originated from her neighbor and that this person played music to bully her. She was convinced that the nurses at the nursing home did not intervene because the neighbor was rich, and she was also suspicious of the food after experiencing diarrhea. Despite explanations from her direct environment that the sounds were hallucinations and could actually be attributed to the brain, she could not be persuaded. Her medical history comprised diabetes mellitus type 2, hypertension, and myocardial infarction. Her medication upon clinical admission consisted of pantoprazole, insulin, simvastatin, perindopril, metoprolol, lercanidipine, glimepiride, ezetimibe, cholecalciferol, and aspirin. None of these medications could be held responsible for the presence of hallucinations and delusions. Blood tests indicated no abnormalities. The psychiatric examination indicated an impaired short-term memory, word-retrieval difficulties, apraxia, and paranoid delusions. An additional neurological examination was unremarkable, and her brain MRI showed multiple white-matter lesions, mainly in the supratentorial region, not unusual for her age. The EEG showed a diffuse disturbance in the posterior half of the left hemisphere and bilateral temporal disturbances. Epileptiform abnormalities were sporadically present, not correlated to the presence of the hallucinations. During admission she scored 22/30 points on the MMSE, suggesting cognitive impairment, and she was accordingly diagnosed with mixed-type dementia (Alzheimer’s and vascular dementia). This was also identified as the probable cause of the hallucinations and delusions. During admission it remained difficult to augment her paranoid delusions. Although antiepileptics were considered by her treating physician because of the EEG results, it was decided to target the psychotic symptoms with risperidone 1.5 mg daily. The musical hallucinations remained present, but they became tolerable and the patient gained insight into her situation; the delusions faded, and she felt ashamed of the false accusations that she had made.

### 3.2. Patient 2

In 2013, a 90-year-old woman with progressively impaired hearing over a course of 15 years was admitted to Parnassia Psychiatric Institute for the diagnostics and treatment of musical hallucinations with secondary paranoid beliefs. She could not remember when the hallucinations had started. She heard fragments and entire pieces of music, consisting of a male voice accompanied by a piano. She recognized the music, but could not recall any titles. The hallucinations were present daily for about 12 h. They receded into the background when she turned on the television or had visitors, and increased when she was alone. She wore hearing aids on both sides which had no effect on the hallucinations. Despite being perceived inside her head, the music exhibited a distinct perceptual quality to the patient. She occasionally regarded the hallucinations as bothersome, particularly because they interfered with her conversations and hindered her ability to concentrate. She was convinced that the singing came from a male neighbor on a different floor of her apartment complex, but her neighbor next door did not hear anything. She called the police twice, but they did not hear the music either. On May 4, Remembrance Day, she became angry because the male voice continued to sing during the 2 min of silence. Once admitted to our hospital, she also harbored paranoid beliefs that the nursing staff were attempting to harm her with the medications they administered, and that they blew warm air currents in her direction as a form of harassment. On one occasion she also experienced visual and tactile hallucinations, but these did not occur simultaneously with the musical hallucinations. Her medical history comprised cardiac arrhythmia, and her psychiatric history consisted of personality disorder not otherwise specified and minor depressive disorder (HDRS score 4). Her medication consisted of esomeprazole, propranolol, raloxifene, oxazepam, lormetazepam, and calcium carbonate, which were not held responsible for the musical hallucinations and delusions. Blood tests were unremarkable. The brain CT scan showed moderate to severe atrophy and severe, mostly confluent white-matter damage. She refused to undergo a brain MRI and EEG. She scored 24/30 on the MMSE, suggesting cognitive impairment, and during admission she was diagnosed with vascular dementia, which was held to be the cause of the musical hallucinations. Upon prescription of risperidone 1 mg a day, the musical hallucinations and secondary delusions diminished within a week. The hallucinations remained present only to a mild extent and eventually the delusions went into complete remission.

### 3.3. Patient 3

In 2014, a 78-year-old woman with hearing impairment was referred to the outpatient department of Parnassia Psychiatric Institute for an assessment of her musical hallucinations. She had been experiencing these for over a year. Initially, she heard Christmas carols continuously; later on, the music became unrecognizable to her. She was convinced that her neighbors were playing music on a loop, even when they were not at home. She got so angry that she wanted to harm the neighbors (yet she refrained from taking any actual action), and became estranged from her sister who maintained that she did not hear the music. Despite also hearing it when she stayed at her daughter’s house, she still believed that it originated from the neighbors at home. Adjusting her hearing aids did not help, but watching television reduced the volume of the hallucinations. She described them as very disturbing and loud, scoring them as 8–9/10 on a visual analog scale (VAS), and stated that the noise interfered with her ability to fall asleep. Due to the hallucinations she also experienced difficulty with concentrating. The patient had been living alone for 20 years, and hardly spoke to anyone. Her medication comprised co-codamol, simvastatin, metformin, gliclazide, sitagliptin, and atenolol. Although she had had no previous mental complaints, the musical hallucinations made her depressed, scoring 9 points on the HDRS. The mirtazapine prescribed by her family physician hardly helped. The results of the blood tests are missing. A neurological and psychiatric assessment showed no other abnormalities than the mood changes. In the consulting room, the hallucinations began after 30 s of silence. The patient scored 79 out of 100 on the Addenbrooke’s Cognitive Examination-Revised (ACE-R), suggesting cognitive impairment. A brain MRI showed white-matter changes consistent with age. A few days after starting rivastigmine 1.5 mg daily, the hallucinations became less loud and less frequent (VAS 5/10), and her depressive mood and anger vanished. The patient gained more insight into the origin of the music even though at times she still suspected the neighbors. Three months into treatment, when seen in the consulting room, the music started after 1 min of silence. The rivastigmine dose was increased to 4.5 mg daily in an attempt to treat the musical hallucinations and cognitive impairment, but no follow-up data was available due to the patients’ death half a year later because of pneumonia.

### 3.4. Patient 4

In 2018, a 75-year-old woman with impaired hearing was referred to Parnassia Psychiatric Institute because of musical hallucinations which she had been experiencing since she had received hearing aids 2 years earlier. She perceived fragments of popular pop songs inside and outside her head, which she could not specify. The patient responded to the hallucinations by shouting at her neighbors from within the living room, urging them to be quiet. Despite her discomfort she did not dare to confront them personally. The patient was unable to recall the average frequency and duration of her hallucinations, but during the daytime they only receded into the background when she carried out tasks that required her full attention or when she spoke to people. She occasionally found the hallucinations bothersome, especially because they interfered with her falling asleep. Of note, the patient also perceived laser beams on the ceiling, as well as strange images (possibly complex visual hallucinations) and visual distortions (metamorphopsias). Her past psychiatric history consisted of bipolar II disorder, and her medical history comprised Alzheimer’s disease, hyperthyroidism, hypertension, varices, and breast cancer. Her medication comprised lithium, valproic acid, nortriptyline, and rosuvastatin. Blood tests showed no abnormalities. A brain MRI showed white-matter changes consistent with age. The patient refused to have an EEG performed. Upon prescription of risperidone 1 mg daily the hallucinations went into complete remission. It is unclear whether the antipsychotic had any effect on the delusions. After 5 years, the risperidone was discontinued because of passivity, and the musical hallucinations did not return.

### 3.5. Patient 5

In 2019, a 74-year-old woman with cognitive impairment and impaired hearing was referred to Parnassia Psychiatric Institute because of musical hallucinations which she had experienced for 2 years. In her backyard and on the streets she heard a variety of singers performing both old and recent Dutch music (including the classic children’s song “*Sinterklaas Kapoentje*”). She was convinced that the singers were surrounded by public. The patient perceived the music from different directions outside her head and she heard it through both ears. It was present continuously, from the moment she awoke until late at night. The patient experienced them as troublesome, particularly because they hindered her ability to sleep properly. She was convinced that her neighbors were causing the noise, despite reassurances from her family that they could not hear it, which created conflicts at home. According to her husband, the patient frequently placed her ear against the wall to listen to the music. She moreover called the police on numerous occasions, and wanted to collect signatures from others to make a statement against the perpetrators. The general practitioner prescribed haloperidol drops, which her husband secretly poured into her drink (1 mg daily). This had no effect on the hallucinations, delusions, and lack of insight. She had hearing aids, but refused to use them. Her somatic history comprised right-sided vestibular schwannoma, diabetes mellitus type 2, hypertension, macular pucker in the right eye, and monocular blindness in the left eye. Her medication consisted of metformin and simvastatin. Blood tests were unremarkable. She scored 13/30 on the MMSE, suggesting cognitive impairment and the brain MRI showed multiple white-matter lesions and a small right-sided vestibular schwannoma. She refused to have an EEG performed. The advice was to prescribe rivastigmine, but the patient was lost to follow-up and it is unclear whether she ever took this medication.

## 4. Discussion

All five patients here described experienced musical hallucinations, paranoid delusions, and lack of insight. It is postulated that the delusions were secondary to the musical hallucinations based on the history provided by the patient and collateral sources. The development of delusions subsequent to the onset of hallucinations, as well as the specific focus of the delusions on the hallucinatory experiences, support this hypothesis. All patients perceived the hallucinations as bothersome, particularly because they interfered with conversations, concentration and the ability to fall asleep. Also, the musical hallucinations and secondary delusions had severe social consequences, such as frequently calling the police, and conflicts with relatives and neighbors, which corresponds with the social repercussions described by Coebergh et al. (15). All patients were elderly women, had impaired hearing, and suffered from cognitive impairment, likely neurodegenerative. EEG findings, insofar as they were available, did not provide any clues to other mechanisms underlying the hallucinations. Cognitive impairment can be accompanied by white-matter damage (24), but in the case of the patients, the white-matter damages fell within the boundaries of normal in their relation to age. Therefore, we could not establish a link between white-matter damage and the occurrence of musical hallucinations *per se*. We believe that the combination of factors mentioned above was responsible for the rare clinical presentation. In what follows, we will discuss the contribution of each individual factor in some more detail, and attempt to outline the pathophysiological mechanisms involved.

### 4.1. Patient characteristics

As already noted by Berrios (1), the prevalence of musical hallucinations is higher in females compared to males. A similar overrepresentation of females was found among people with vascular dementia and hallucinations in general (25), and, in people with hearing impairment, for musical and other auditory hallucinations (6). It is unclear whether this overrepresentation is due to underreporting in men, or perhaps to an as yet unidentified risk factor in women. As noted above, advanced age is also a known risk factor for musical hallucinations. Therefore, it is plausible that neither female gender nor advanced age are responsible for the delusions and lack of insight encountered in our five patients.

### 4.2. Hearing impairment

Hearing impairment, whether or not in combination with tinnitus, is yet another known risk factor for musical hallucinations. The underlying mechanism is believed to be deafferentation due to sensory deprivation, i.e., a situation where a lack of incoming sensory input to the auditory system leads to spontaneous neural activity in the network responsible for the processing of music. In analogy with the visual hallucinations characteristic of Charles Bonnet syndrome, this variant of musical hallucinosis has been previously referred to as the auditory Charles Bonnet syndrome (12). The reason why musical hallucinations rather than other types of auditory hallucination are thus produced is unclear, but the parasitic memory hypothesis suggests that this may be due to our general inability to forget musical pieces that have made an impression on us. First proposed by Crick and Mitchison (26), the parasitic memory hypothesis starts from the premise that we are fundamentally incapable of dealing with the bombardment of stimuli that we habitually undergo during our waking lives, and that dreaming may have a function in removing unnecessary or unwanted memory traces. The hypothesis was first applied to the topic of musical hallucinations by Keshavan et al. (27), who suggested that musical memories, and especially those which arouse affect, may be resistant to the type of “unlearning” described by Crick and Mitchison (26). As a consequence, these memories may resonate in the widely distributed brain network devoted to music, and take the form of hallucinations when regular auditory input is diminished. The parasitic memory hypothesis may thus serve as an explanation for the mediation of musical hallucinations in the hearing impaired, although it does not explain the paranoia and lack of insight also encountered in our five patients. Since hearing impairment itself is also associated with an increased risk of delusions (28), impaired hearing may be yet another contributing factor to the combination of musical hallucinations and delusions. It remains the question, however, which additional risk factors contribute to this comorbidity, since not all hearing-impaired people with musical hallucinations go on to develop secondary delusions and many of them briefly attribute the music to external sources, but quickly gain insight.

### 4.3. Paranoia

People with paranoid delusions or a preexistent paranoid personality are prone to a lack of insight into the nature and origin of their hallucinations (29). As a consequence, they may react to these perceptual phenomena with paranoid delusions, too. However, the described patients had previously never been diagnosed with a psychotic disorder, except for patient 4, the 75-year-old woman earlier diagnosed with a bipolar II disorder with psychotic features. More than 50% of the people with bipolar disorder experience psychotic symptoms at some point, although mainly during manic episodes (30). Even though our patient was not seen by us during a manic episode, she did experience additional perceptual symptoms in the form of visual hallucinations and metamorphopsias. There were moreover indications of suspicion in her case, as she scored points on the suspiciousness subscale and the social anxiety subscale of the SPQ. This might suggest a psychotic episode in the context of bipolar disorder, and thus explain the combination of musical hallucinations and secondary delusions in her case. Alternatively, the delusions may have been secondary to deficient reality-monitoring strategies (31).

### 4.4. Cognitive impairment

Apart from psychosis, one of the best-known mechanisms that may lead up to deficient reality monitoring is cognitive impairment, a condition that all of our five patients suffered from. Studies have found that people with dementia and other neurocognitive disorders often have a reduced awareness of their cognitive deficits, and may struggle with introspection and self-awareness (32, 33). This lack of insight may extend to their musical hallucinations, making it difficult for them to recognize these perceptions as hallucinatory in nature, and encouraging secondary delusions. This was also the case with two of the three patients mentioned in the introduction, who likewise reacted to their musical hallucinations with paranoid delusions (18, 19). There are also published cases where neurodegenerative disorders such as Alzheimer’s disease and Lewy body dementia led up to a misattribution of the origin of musical hallucinations [e.g., (19, 34)]. Of note, three other participants to our study had an MMSE just below 25, which is generally considered the cut-off score for mild cognitive impairment. Since these three patients had not gone on to develop paranoid delusions, the relationship between paranoid delusions and cognitive impairment was not 1:1. Another possible explanation for lack of insight (which is not directly applicable to our patients) is the emotional impact that musical hallucinations tend to have, which may be more salient than the perceptual abnormality itself (35). In such cases people may be focused more on the emotional response elicited by the music than on the issue of an internal or external source.

### 4.5. Pathophysiology

Together, the factors detailed above may have contributed to the rare combination of musical hallucinations, secondary delusions, and lack of insight in our patients. In this multifactorial process, female gender and older age may have acted as general risk factors, along with vulnerability to psychosis and hearing impairment. Especially in combination with social isolation – causing a further reduction in auditory input – the latter factor may contribute to deafferentation and the subsequent recruitment of internally mediated auditory percepts. The parasitic memory hypothesis provides a plausible explanation why these percepts took the form of musical hallucinations, especially in these patients with cognitive impairment. After all, musical memories are resistant to unlearning, and may be retained even after other types of memory have been compromised. Cognitive disorders have been mentioned before as risk factors for musical hallucinations (14), and in his book *Musicophilia*, Sacks (36) gives several examples of severely demented people who were able to skillfully sing or play large musical repertoires from the past. As to the lack of insight that our patients showed into the origin of the music they perceived, and the subsequent development of paranoid delusions, cognitive impairment probably played a crucial role as well. This is in line with a study by Golden and Josephs (35), who noted that people with neurocognitive disorders and musical hallucinations often lack insight into their hallucinations. Contrary to the majority of people who experience musical hallucinations, our patients showed substantially impaired reality monitoring skills, which we attribute primarily to their cognitive decline.

### 4.6. Treatment results

Finally, there may be some lessons to be learned from the response to treatment. Three of the described patients were treated with risperidone, an atypical antipsychotic which – at least in the lower dosages prescribed in these cases – yields fewer extrapyramidal side effects than first-generation antipsychotics tend to do. Since risperidone has minimal anticholinergic properties, it is also considered useful in the treatment of elderly patients with dementia with psychotic features (37–39). Although the treatment of musical hallucinations with the aid of antipsychotics is generally considered ineffective, or moderately effective at best (15, 40), in these three patients, risperidone had a positive effect on the hallucinations *and* secondary delusions. This corresponds with a review by Sink et al. (41) on the effects of pharmacological treatment in patients with dementia and adjuvant neuropsychiatric symptoms. Therefore, a preliminary conclusion may be that the treatment of musical hallucinations and secondary delusions with antipsychotics may be most beneficial in patients with cognitive impairment. Of note, however, nowadays the black box warning of risperidone cautions against the use of this drug in elderly persons with dementia. This is based on the finding that people in this group have a slightly higher mortality rate than those using a placebo. For example, Haupt et al. (42) found this rate to be 0.9%. Since all antipsychotics have their own idiosyncratic side-effect profile, this may not be a pressing reason to withhold risperidone from people in this group suffering from musical hallucinations. Nonetheless we consider exploring the efficacy and safety of other antipsychotics for this group an important goal to pursue. If non-vascular neurodegenerative cognitive impairment is present it would also be reasonable to first start treatment with acetylcholinesterase inhibitors or related medications (40, 43), and only start treatment with antipsychotics in non-responsive cases. However, all of this is in need of replication in future studies, preferably with larger numbers of participants.

### 4.7. Limitations

A limitation of the present study is that the number of patients included was very small. This is not surprising in the light of the relative rarity of musical hallucinations. Secondly, we need to point out that the *MuHa Questionnaire* is a non-validated, semi-structured questionnaire that was not independently applied by more than one person in our study. Instead, each member of the team was trained by more experienced members to administer it, and if there were any questions or inconsistencies these were discussed with the PI (JDB). Thirdly, the explanations that we provide for the association between musical hallucinations, secondary delusions, and lack of insight are based on theoretical insights rather than empirical testing. Fourth, even though medication was not held responsible for the onset of the musical hallucinations in our patients, these hallucinations have occasionally been described in the context of antidepressant use (44), high salicylate levels (45), and the use of beta blockers (46). Fifth and finally, due to a few missing data two of our five case descriptions were not entirely complete. Therefore, a somatic cause could not be ruled out entirely in these cases, even though it was not suspected clinically.

## 5. Conclusion

The majority of people who experience musical hallucinations do not go on to develop secondary delusions. Nonetheless, we found five patients in a group of 81 (6%) who lacked insight and developed paranoid delusions in reaction to the music they perceived. All of them perceived the hallucinations as bothersome and the combination of hallucinations and delusions had severe social consequences. All patients were female, of advanced age, and hearing-impaired, and all of them were diagnosed with cognitive impairment. We provided a tentative reconstruction of the multifactorial process that may have led up to this rare and debilitating combination of symptoms, and singled out cognitive impairment as the most likely contributing factor to the secondary delusions and lack of insight. Although musical hallucinations in general do not tend to respond well to antipsychotics, three out of the five patients described here (60%) were given risperidone, with successful outcomes in all three cases. The use of hearing aids was of no avail against the hallucinations. Further research is needed to elucidate the underlying mechanisms of musical hallucinations, secondary delusions, and lack of insight, and to develop evidence-based treatment methods for people who experience this triad of symptoms. In the light of the black box warning for risperidone, an exploration of the efficacy and safety of other antipsychotics for this group is strongly advised.

## Data availability statement

The raw data supporting the conclusions of this article will be made available by the authors, without undue reservation.

## Ethics statement

The studies involving humans were approved by the Ethical Review Board of the Haga Hospital, The Hague, Netherlands. The studies were conducted in accordance with the local legislation and institutional requirements. The participants provided their written informed consent to participate in this study. Written informed consent was obtained from the individual(s) for the publication of any potentially identifiable images or data included in this article.

## Author contributions

MB drafted the manuscript, acquired data for the case descriptions, revised the manuscript, gave final approval of the version to be published, and agreed to be accountable for all aspects of the work in ensuring that questions related to the accuracy or integrity of any part of the work are appropriately investigated and resolved. RL, OB, and ML acquired data for the case descriptions, revised the manuscript, gave final approval of the version to be published, and agreed to be accountable for all aspects of the work in ensuring that questions related to the accuracy or integrity of any part of the work are appropriately investigated and resolved. JAC and JDB contributed to the conceptualization and design of the study, collected data, contributed to the interpretation of findings, revised the manuscript, provided approval for the final version to be published, and took responsibility for the integrity of the work by actively addressing and resolving any questions related to accuracy or integrity on any part of the study. All authors contributed to the article and approved the submitted version.
