# Supplementary material for: Musical hallucinations, secondary delusions, and lack of insight: results from a cohort study
Source: Front Psychiatry. 2023 Sep 28;14:1253625. doi: 10.3389/fpsyt.2023.1253625 (PMC10569219; doi:10.3389/fpsyt.2023.1253625)
Supplement: Supplementary file 1 [file Data_Sheet_1.PDF]

MuHa Questionnaire on the natural history, treatment and phenomenology of musical hallucinations

Questionnaire developed by Drs. J.A. Coebergh[1,2,3], Prof. dr. I.E.C. Sommer[4] and Dr. J.D. Blom[5,6]
Version 1.0 Copyright 2010 by the authors
REC Nr 13/LO/1586

1 Department of Neurology, Haga Hospital, The Hague, the Netherlands
2 Department of Neurology, Ashford/St. Peter's Hospital, Chertsey, UK
3 Department of Neurology, St George's Hospital, Tooting, UK
4 Brain Division, UMC Utrecht, Utrecht, the Netherlands
5 Parnassia Psychiatric Institute, The Hague, the Netherlands
6 Department of Psychiatry, University of Groningen, Groningen, the Netherlands

Patient Name:
Date of Birth:
File number:
Name of researcher:
Date of research:
Date of MRI:
Date on which the musical hallucinations began:
Number of years since onset:
Measurement number: 1/2/3/4/5

1 Type of music (open question)
What musical hallucinations have you heard for the past month? (Please write in detail: interrogate anthem, tearjerkers, carols, hymns, nursery rhymes, classical music, etc.) and is there delay in onset after external stimulation?

2 Quality of hallucinations
2.1 Does the music sound pure?
- O Yes
- O No
- O Sometimes pure, sometimes false

2.2 Do you hear complete songs?
- O Yes
- O No, only fragments or fragments
- O Both

2.3 Is the same music ever repeated?
O Yes, maximum consecutive times…
O No

2.4 Do you hear music you've previously heard?
O Yes, songs or music that I used to know
O Yes, songs or songs that I have just heard
O Other, ...
O No, I hear .....

2.5 How do you hear it?
O Through my ears, both sides
O Through my left ear
O Through my right ear
O Inside my head
O Both inside and outside my head
O Other, ...
O Do not know

2.6 From what direction does the music come?
O From the right side
O From the left side
O Straight from the front
O Right behind
O Above my head
O Below
O From the inside
O Other, ...
O Do not know

2.7 Is the type of music congruent with your mood?
O Yes, because .....
O No
O Other, ...
O Do not know

3 Frequency
3.1 On how many days of the past month have you experienced musical hallucinations?
1/2/3/4/5/6/7/8/9/10/11/12/13/14/15/16/17/18/19/20/21/22/23/24/25 / 26/27/28/29/30/31

3.2 How many hours per day on average?
1/2/3/4/5/6/7/8/9/10/11/12/13/14/15/16/17/18/19/20/21/22/23/24

3.3 What number would you give between 0 (never) and 10 (continuous?)
0/1/2/3/4/5/6/7/8/9/10

4 Control

4.1 Can the musical hallucinations be influenced by distraction?
- O Yes, namely .... The hallucinations are then .....
- O No
- O Do not know

4.2. Do you consciously control the hallucinations?
- O Yes, namely .... The hallucinations are then .....
- O No
- O Do not know

4.3 Do you influence the choice of music / songs?
- O Yes, namely .... The hallucinations are then .....
- O No
- O Do not know

4.4 Do you ever sing along with musical hallucinations?
- O Yes
- O No

4.5 Does it help to sing along with musical hallucinations?
- O Yes, the hallucinations are then .....
- O No

4.6 Do you have influence in other ways on the musical hallucinations?
- O Yes, namely .....
- O No

5 Effects on functioning

5.1 Are your musical hallucinations a nuisance/hindrance/annoyance?
- O Yes
- O No
- O Sometimes, sometimes not

5.2 Do they disturb you at bedtime?
- O Yes
- O No
- O Sometimes, sometimes not

5.3 Do they disturb you in conversation?
- O Yes
- O No
- O Sometimes, sometimes not

5.4 Do they disturb you in concentrating?
- O Yes
- O No
- O Sometimes, sometimes not

5.5 Do they make you feel like you are mentally ill?
- O Yes

O No
O Sometimes, sometimes not

5.6 Do they disturb you in another way?
O Yes, namely ....
O No

6 Influence of medication
6.1 What medications are you currently using?

Drug 1:	Duration of use:	Effect:
Drug 2:	Duration of use:	Effect:
Drug 3:	Duration of use:	Effect:
Drug 4:	Duration of use:	Effect:
Drug 5:	Duration of use:	Effect:

6.2 Does your medication help against the hallucinations?
O Yes, Drug (1/2/3/4/5)
O No
O Sometimes, sometimes not
O Do not know

6.3 Which influence has drug (x) on the musical hallucinations?
Repeat the question for any drug which affects the hallucinations!
O They disappeared
O They sound less loud
O They sound further away
O I hear them less often
O The quality of the music changes, namely, ...
O I have more influence on them
O They bother me less
O Other, namely .....
O Do not know

6.4 What medications have you tried in the past against the musical hallucinations? For how long? And to what effect?

Drug 1:	Year:	Duration of use:	Effect:
Drug 2:	Year:	Duration of use:	Effect:
Drug 3:	Year:	Duration of use:	Effect:
Drug 4:	Year:	Duration of use:	Effect:
Drug 5:	Year:	Duration of use:	Effect:

6.5 Have you tried other treatments against the musical hallucinations?
O Yes, namely .....
O No

7 Hallucinations last month
7.1 Have the musical hallucinations in the past month changed in severity?
O Yes, the severity has increased
O Yes, the severity decreased
O No, not changed

O Do not know

7.1.1 Please indicate on a scale of 0 to 10 (0 = no discomfort, 10 = very severe) the severity of hallucinations in the past month and how it has developed (Indicated by an arrow, for example, from 3 to 5)

0/1/2/3/4/5/6/7/8/9/10

7.2 Have the musical hallucinations in the past month changed in frequency?

O Yes, the frequency is increased
O Yes, the frequency is decreased
O No, not changed
O Do not know

7.2.1 Please indicate on a scale of 0 to 10 (0 = never, 10 = always) how the frequency of hallucinations in the past month has developed (Indicated by an arrow, for example, from 3 to 5)

0/1/2/3/4/5/6/7/8/9/10

7.3 Have the musical hallucinations in the past month changed in volume?

O Yes, the volume has increased
O Yes, the volume has decreased
O No, not changed
O Do not know

7.3.1 Please indicate on a scale of 0 to 10 (0 = quiet, 10 = deafening) how the volume of hallucinations in the past month has developed (Indicated by an arrow, for example, from 3 to 5)

0/1/2/3/4/5/6/7/8/9/10

8 Hearing

8.1 Do you suffer from tinnitus?

O Yes, left since .....
O Yes, right since .....
O Yes, both sides since.....
O No
O Sometimes, sometimes not

8.2 Do you suffer from hearing loss?

O Yes, left since .....
O Yes, right since .....
O Yes, both sides since.....
O No

8.3 Is the hearing loss stable?

O Yes
O No, it worsens
O No, it improves

8.4 Do you have a hearing aid?
- O Yes, since .....
- O No

8.5 If you have a hearing aid: do you use this?
- O Yes
- O No
- O Sometimes, sometimes not (explanation: ....)

8.6 If you have a hearing aid: does this affect the musical hallucinations?
- O Yes, they are less loud
- O Yes, I hear them less frequently
- O Yes, they bother me less
- O Sometimes, sometimes not
- O Other, ...

9 Hallucinations in other sensory modalities

9.1 Have you ever had other types of hallucination?
- O Images, visions
- O Deformations of normal observed objects
- O Smells
- O Strange taste
- O Unexplained touch on the body
- O Unexplained feelings in the body
- O Sexual hallucinations
- O Strange temperature
- O Feeling as if my body or body parts move
- O Feeling like a body part has another position
- O Other, namely…

9.2 Did these occur simultaneously with musical hallucinations?
- O Yes, along with the musical hallucinations
- O Sometimes simultaneously, sometimes alternating
- O No

9.3 Have you in the past month had other types of hallucinations?
- O Images, visions
- O Deformations of normal observed objects
- O Smells
- O Strange taste
- O Unexplained touch on the body
- O Unexplained feelings in the body
- O Sexual hallucinations
- O Strange temperature
- O Feeling as if my body or body parts move
- O Feeling like a body part has another position
- O Other, namely…

9.4 Did these occur simultaneously with musical hallucinations?

O Yes, along with musical hallucinations
O Sometimes simultaneously, sometimes alternating
O No

10 Musicality
10.1 Are you musical?
O Yes (explanation: singing, instrument playing, composing, etc.?)
O No

10.2 Do you suffer from amusia?

O Yes (explanation: recognition of higher / lower frequencies/ tone / melody / emotional charge; inability to write music, whistle, sing, read, recognize, play music)
O No

11 Concluding questions
11.1 Are there other health problems that may affect the musical hallucinations?
O Yes, because .....
O No

11.2 Social isolation: Do you get help from family, neighbours or home support?

O Yes (note: ....)
O No (note: ....)

11.3 Loneliness: Do you feel lonely or even very lonely?

O Yes (note: ....)
O No (note: ....)

11.4 Are there other things that are important for this research?
O Yes, because .....
O No

11.5 Do you have any questions?
